# A comparative study on parental rearing styles and competitive attitudes among college students from different family income backgrounds

**DOI:** 10.3389/fpsyg.2025.1597721

**Published:** 2025-06-24

**Authors:** Shuqing Zhou, Dong Wang, Tingting Xu

**Affiliations:** School of Public Health, Shandong Second Medical University, Weifang, China

**Keywords:** family income, parental rearing style, competitive attitude, mental health, family environment

## Abstract

**Objective:**

To explore the differences in parental rearing styles and competitive attitudes between college students from low-income and non-low-income families. The goal is to provide insights into their holistic development and psychological adaptation in diverse competitive environments.

**Methods:**

A total of 1,000 college students were surveyed using a general information questionnaire, the *Egna Minnen Beträffande Uppfostran* questionnaire, and the Competitive Attitude Scale. Among them, 188 were identified as low-income students and 750 as non-low-income students.

**Results:**

(1) Significant differences were observed between low-income and non-low-income students in parental emotional warmth and understanding, paternal denial and rejection, maternal favoritism, and malignant competitive attitude (*p* < 0.05). (2) Parental emotional warmth and understanding were positively correlated with benign competitive attitude (*p* < 0.01). (3) Parental rejection, denial, favoritism, and overprotection were negatively correlated with malignant competitive attitude (*p* < 0.05). (4) Paternal punishment and strictness were positively correlated with malignant competitive attitude (*p* < 0.01).

**Conclusion:**

Positive parental rearing styles enhance the subjective well-being of low-income students and foster benign competitive attitudes, whereas negative parental rearing styles reduce well-being and promote excessive competitive tendencies.

## 1 Introduction

Parental rearing style is closely associated with individual psychological development, and its impact on competitive attitude has become a research focus in educational and psychological fields. Studies indicate significant correlations between parental rearing styles and children’s competitive attitudes (Ding et al., 2024). The influence of fathers and mothers differs markedly in the upbringing process (Yang and Zhao, 2020). Parental rearing styles can be categorized as positive or negative based on dimensions such as emotional warmth, strictness, and overprotection (Žerak et al., 2024). Investigating the relationship between parental rearing style and competitive attitude helps reveal how family environments shape psychological traits, providing theoretical support for optimizing family education. Additionally, family income, as a critical contextual variable, significantly influences parental rearing styles and individual psychological development (Afif, 2019).

Existing research on psychological development predominantly focuses on adolescents, exploring links between parental rearing styles and academic performance or mental health (Boonk et al., 2018), while studies on college students remain limited. College students represent a unique population within the developmental stage of emerging adulthood. Significant psychosocial distinctions from adolescence include: intensified needs for autonomy, novel challenges in career orientation and independent decision-making (Arnett, 2000), and transformed familial influence shifting from direct control to implicit guidance (Arnett, 2014). This study therefore targets the collegiate cohort to unravel the mechanistic pathways linking family income, parental rearing styles, and competitive attitudes during this pivotal developmental window. Most prior work examines single variables, lacking a comprehensive analysis of the interplay among family income, parental rearing style, and competitive attitude. This study addresses these gaps by focusing on the college student population, analyzing the relationships among family income, parental rearing style, and competitive attitude. It reveals differences in rearing styles and competitive attitudes between low-income and non-low-income students and explores their correlations.

This study fills a critical research void by targeting college students as the research subject. It provides a novel perspective for understanding the psychological adaptation mechanisms of college students by holistically examining the connections among family income, parental rearing style, and competitive attitude. Findings from relevant literature (Luo, 2022; Ge et al., 2022; Ju et al., 2020) indicate significant differences in competitive attitudes between low-income and non-low-income students. These disparities arise from multifaceted causes, with the parental rearing style in their families of origin playing a central role. Parental rearing styles subtly shape psychological development through daily interactions and emotional transmission, thereby influencing competitive attitudes. Low-income students may exhibit high levels of anxiety, depression, and inferiority due to economic pressures and distinct rearing practices in their families of origin. These psychological traits further impact their competitive attitudes.

## 2 Methods

### 2.1 Participants

Participants were selected using the university’s financial aid database, which identified low-income students. A total of 188 low-income students and 750 non-low-income students were screened. Stratified random sampling was then employed to recruit 1,000 participants, ranging from freshmen to seniors, across two universities in Weifang. A total of 938 valid questionnaires were collected, yielding a response rate of 93.8%. During data collection, a behavioral quality control model was applied to evaluate the sincerity of respondents’ answers, identify potential batch responses from bots or individuals, and flag low-quality or invalid questionnaires. Participants’ response quality was recorded to refine future survey sampling. To address sample size imbalance, we implemented stratified random sampling with post-stratification weighting coefficients calculated using Cochran’s formula. This procedure effectively minimized selection bias, establishing measurement invariance (χ^2^/df < 2, CFI > 0.95) for cross-group behavioral comparisons. The purpose of the study was not disclosed in advance to mitigate emotional resistance caused by the concentrated testing of low-income students.

### 2.2 Instruments

#### 2.2.1 General information questionnaire

The general information questionnaire included basic information such as age, gender, place of origin, department, academic year, family economic status, and single-parent family status.

#### 2.2.2 Parental rearing style

Parental rearing style refers to a relatively stable behavioral pattern encompassing various parenting behaviors during the process of raising and educating children (Perris et al., 1980). This study utilized the Chinese version of the *Egna Minnen Beträffande Uppfostran* (EMBU-C) questionnaire, originally developed by Perris et al. (1980) and revised by Yue et al. (1993). The EMBU comprises 66 items, requiring participants to retrospectively evaluate the rearing styles of their fathers and mothers. It includes six paternal factors (PF) and five maternal factors (MF): PF1: paternal emotional warmth and understanding; PF2: paternal punishment and strictness; PF3: paternal over-interference; PF4: paternal rejection and denial; PF5: paternal favoritism toward the participant; PF6: paternal overprotection, MF1: maternal emotional warmth and understanding; MF2: maternal punishment and strictness; MF3: maternal over-interference and overprotection; MF4: maternal rejection and denial; MF5: maternal favoritism toward the participant. The revised version of this scale demonstrated good reliability and validity. In this study, the overall Cronbach’s α coefficient for the scale was 0.79. The average Cronbach’s α coefficients for individual factors ranged from 0.65 to 0.87 (mean = 0.71). Split-half reliability coefficients ranged from 0.61 to 0.91 (mean = 0.79), and test–retest reliability coefficients (2-week interval, *n* = 186) ranged from 0.66 to 0.90 (mean = 0.76, all *p* < 0.001), confirming the robust reliability of the scale (Rademacher et al., 2023).

#### 2.2.3 Competitive attitude

Individual competitiveness refers to the relatively stable psychological characteristics manifested in the competitive awareness, motivation, cognition, and attitude of an individual, representing a comprehensive psychological construct (Masud et al., 2019). Competitive attitude, as a component of individual competitiveness, denotes an intrinsic, stable, and enduring psychological response tendency toward competition. It is commonly conceptualized across two dimensions: benign competition and excessive competition. This study employed the Competitive Attitude Scale (CAS) revised by Chen et al. (2003). The scale comprises two subscales: the Benign Competitive Attitude Subscale and the Excessive Competitive Attitude Subscale, The scale comprises two subscales: the Benign Competitive Attitude Subscale and the Excessive Competitive Attitude Subscale, with a total of 27 items. The CAS exhibits strong reliability and validity, with an internal consistency coefficient (Cronbach’s α) of 0.860 for the Benign Competitive Attitude Subscale and 0.710 for the Excessive Competitive Attitude Subscale.

#### 2.2.4 Definition of low-income students

A student in financial difficulty is one whose financial ability and that of their family make it hard to meet the basic expenses for study and living during the school year. The basis for recognition includes: family economic factors, special group factors, factors of regional economic and social development levels, factors of emergencies, factors of student consumption, and other relevant factors affecting the economic situation of a family. Students with family economic difficulties are classified into three levels: general difficulty, difficulty, and special difficulty. General difficulty means that the student and their family cannot yet fully provide for the student’s study and basic living expenses during the school period, and the monthly living expenses that the family can provide is less than 1,000 yuan; difficulty means that the student and their family can only partially provide for the student’s study and basic living expenses during the school period, and the monthly living expenses that the family can provide is less than 600 yuan; special difficulty means that the student and their family cannot provide for the student’s study and basic living expenses during the school period, and the monthly living expenses that the family can provide is less than 1,000 yuan. Special hardship means that the student and their family are completely unable to provide for the student’s study and living expenses during the school period, and the monthly living expenses provided by his/her family is less than 300 yuan. This study refers to students with financial difficulties who are recognized as being in difficulty and special difficulty during their school years.

### 2.3 Research process and data analysis

Statistical analysis was conducted using SPSS 27.0 software. A series of statistical indicators and analytical methods was applied. Descriptive statistics (e.g., means, standard deviations) were used to summarize the basic characteristics of family income, parental rearing style (including dimensions such as emotional warmth/understanding, rejection/denial, favoritism, overprotection, and punishment), and competitive attitude (benign and malignant competition). Analysis of variance was employed to compare the significance of differences in parental rearing styles and competitive attitudes between low-income and non-low-income students. Pearson correlation coefficients were calculated to analyze linear relationships between parental rearing styles and competitive attitudes.

## 3 Results

### 3.1 Comparison of parental rearing styles between low-income and non-low-income students

As shown in Table 1, low-income students scored significantly lower than non-low-income students in paternal emotional warmth and understanding (*p <* 0.05) and paternal denial and rejection (*p <* 0.05). However, no significant differences were observed in paternal punishment/strictness, over-interference, favoritism, or overprotection. Table 2 reveals that for maternal rearing styles, low-income students scored significantly lower than non-low-income students in maternal emotional warmth and understanding (*p <* 0.05) and maternal favoritism (*p <* 0.05). No significant differences were found in maternal over-interference/protection, denial/rejection, or punishment/strictness.

**Table 1:** Independent samples *t*-test of paternal rearing styles between low-income and non-low-income students (*N* = 938).

Group	Low-income students (mean ± SD)	Non-low-income students (mean ± SD)	*t*-value
PF1	45.65 ± 14.16	46.66 ± 13.53	11.29**
PF2	21.27 ± 7.21	21.54 ± 6.91	3.2
PF3	18.97 ± 5.78	19.10 ± 5.43	1.1
PF4	7.32 ± 4.12	7.52 ± 3.92	5.18*
PF5	7.78 ± 2.90	7.83 ± 2.81	0.96
PF6	9.65 ± 3.29	9.64 ± 3.12	2.21

*p < 0.05, **p < 0.01. PF1, paternal emotional warmth and understanding; PF2, paternal punishment and strictness; PF3, paternal over-interference; PF4, paternal rejection and denial; PF5, paternal favoritism toward the participant; PF6, paternal overprotection.

**Table 2:** Independent samples *t*-test of maternal rearing styles between low-income and non-low-income students (*N* = 938).

Group	Low-income students (mean ± SD)	Non-low-income students (mean ± SD)	*t*-value
MF1	52.73 ± 16.18	53.57 ± 15.34	6.11*
MF2	32.99 ± 10.17	33.17 ± 9.46	0.7
MF3	11.24 ± 4.43	11.23 ± 4.18	0.02
MF4	10.85 ± 4.04	10.88 ± 3.81	0.07
MF5	7.78 ± 4.10	8.04 ± 3.94	8.71**

*p < 0.05, **p < 0.01. MF1, maternal emotional warmth and understanding; MF2, maternal punishment and strictness; MF3, maternal over-interference and overprotection; MF4, maternal rejection and denial; MF5, maternal favoritism toward the participant.

### 3.2 Comparison of competitive attitudes between low-income and non-low-income students

As shown in Table 3, no significant difference was observed in benign competitive attitude between low-income and non-low-income students. However, low-income students exhibited significantly lower scores in malignant competitive attitude compared to non-low-income students (*p* < 0.01).

**Table 3:** Independent samples *t*-test of competitive attitudes between low-income and non-low-income students (*N* = 938).

Group	Low-income students (mean ± SD)	Non-low-income students (mean ± SD)	*t*-value
BCA	55.86 ± 7.01	55.61 ± 7.49	2.3
MCA	28.77 ± 4.34	29.38 ± 4.70	35.93**

*p < 0.05, **p < 0.01. BCA, benign competitive attitude; MCA, malignant competitive attitude.

### 3.3 Impact of single-parent status on parental rearing style and competitive attitude among low-income students

The survey revealed that the proportion of single-parent families among low-income students was significantly higher than among non-low-income students. Accordingly, differences in parental rearing styles and competitive attitudes were further analyzed between single-parent and non-single-parent low-income students. Table 4 indicates that single-parent low-income students scored significantly higher in paternal emotional warmth and understanding compared to non-single-parent low-income students (*p* < 0.05). Moreover, single-parent low-income students scored significantly lower in maternal over-interference/protection (*p* < 0.05), maternal rejection/denial (*p* < 0.05), maternal favoritism (*p* < 0.05), and benign competitive attitude (*p* < 0.05). No significant differences were observed in paternal punishment/strictness, paternal over-interference, paternal rejection/denial, paternal favoritism, paternal overprotection, maternal emotional warmth/understanding, maternal punishment/strictness, or malignant competitive attitude.

**Table 4:** Independent samples *t*-test of parental rearing styles between single-parent and non-single-parent low-income students (*N* = 938).

Group	Single-parent families (mean ± SD)	Non-single-parent families (mean ± SD)	*t*-value
Q1	47.2 ± 13.01	45.65 ± 14.16	2.45*
Q3	10.65 ± 3.42	11.23 ± 4.18	−2.12*
Q4	9.24 ± 3.21	10.88 ± 3.81	−2.11*
Q5	7.62 ± 2.95	8.04 ± 3.94	−2.9*
BCA	53.11 ± 6.18	55.61 ± 7.49	−3.37*

*p < 0.05, **p < 0.01. Q1, paternal emotional warmth and understanding; Q3, maternal over-interference and overprotection; Q4, maternal rejection and denial; Q5, maternal favoritism toward the participant; BCA, benign competitive attitude.

### 3.4 Correlation analysis between paternal rearing style and competitive attitude among low-income students

As shown in Table 5, paternal emotional warmth and understanding exhibited a significant positive correlation with benign competitive attitude (*p* < 0.01). Paternal punishment and strictness (*p* < 0.01), paternal rejection and denial (*p* < 0.01), and paternal favoritism toward the participant (*p* < 0.01) were significantly positively correlated with malignant competitive attitude. Paternal overprotection also showed a significant correlation with malignant competitive attitude (*p* < 0.05). However, paternal over-interference demonstrated no significant correlation with either benign or malignant competitive attitude.

**Table 5:** Correlation analysis between paternal rearing styles and competitive attitudes (*r*-values, *N* = 938).

Group	PF1	PF2	PF3	PF4	PF5	PF6
BCA	0.174**	−0.080	0.064	0.022	−0.097	0.018
MCA	−0.099	0.166**	−0.031	0.217**	0.210**	0.121*

*p < 0.05, **p < 0.01. PF1, paternal emotional warmth and understanding; PF2, paternal punishment and strictness; PF3, paternal over-interference; PF4, paternal rejection and denial; PF5, paternal favoritism toward the participant; PF6, paternal overprotection; BCA, benign competitive attitude; MCA, malignant competitive attitude.

### 3.5 Correlation analysis between maternal rearing style and competitive attitude among low-income students

As shown in Table 6, maternal emotional warmth and understanding was significantly correlated with benign competitive attitude (*p* < 0.01). Maternal over-interference and overprotection (*p* < 0.01), maternal rejection and denial (*p* < 0.01), and maternal favoritism toward the participant (*p* < 0.01) exhibited significant negative correlations with malignant competitive attitude. Maternal emotional warmth and understanding also showed a negative correlation with malignant competitive attitude (*p* < 0.05). Maternal over-interference and overprotection (*p* < 0.01) and maternal rejection and denial (*p* < 0.01) were significantly positively correlated with malignant competitive attitude. Maternal punishment and strictness demonstrated no significant correlation with either benign or malignant competitive attitude.

**Table 6:** Correlation analysis between maternal rearing styles and competitive attitudes (*r*-values, *N* = 938).

Group	MF1	MF2	MF3	MF4	MF5
BCA	0.163**	−0.049	−0.183**	−0.207**	0.073
MCA	−0.119*	−0.004	0.303**	0.228**	−0.170**

*p < 0.05, **p < 0.01. MF1, maternal emotional warmth and understanding; MF2, maternal punishment and strictness; MF3, maternal over-interference and overprotection; MF4, maternal rejection and denial; MF5, maternal favoritism toward the participant; BCA, benign competitive attitude; MCA, malignant competitive attitude.

## 4 Discussion

### 4.1 Differences in parental rearing styles between low-income and non-low-income students

This study demonstrates that low-income students scored significantly lower than non-low-income students in the dimensions of parental emotional warmth and understanding and maternal favoritism toward the participant. These findings align with prior research by Lee (2022) and Jeon and Neppl (2016). Potential explanations include as follows: (1) Parents of low-income students often face substantial financial strain, dedicating most of their time and energy to livelihood sustenance. This situation reduces their availability for emotional engagement with their children, thereby diminishing their capacity to provide emotional warmth and understanding. (2) Parents of low-income students generally have lower educational attainment and are more likely to engage in manual labor. Their parenting practices lack guidance from scientific educational theories, focusing predominantly on meeting children’s material needs while neglecting psychological needs. Since maternal education level can exert independent effects on children’s psychological development through non-economic mechanisms (Lundborg et al., 2022; Davis-Kean, 2005), rather than operating solely through family economic conditions, this study excluded parental education levels from its measurement framework.

Notably, low-income students scored significantly lower in paternal rejection and denial compared to non-low-income students. This result may be attributed to the following: (1) Fathers in low-income families often experience guilt over the family’s financial hardships, which they may internalize as a personal failure. Consequently, they adopt compensatory parenting strategies, minimizing rejection or denial behaviors to meet their children’s demands. (2) Low-income students typically exhibit a heightened awareness of their family’s economic limitations, leading them to avoid making excessive or unreasonable requests. This self-restraint reduces opportunities for parental rejection or denial (Zhang et al., 2017; Ding et al., 2022).

### 4.2 Differences in competitive attitudes between low-income and non-low-income students

Existing research predominantly focuses on the relationship between academic performance and mental health, with limited direct exploration of competitive attitudes. Fontenot et al. (2019) highlights that family economic status significantly impacts the psychological and behavioral development of students, suggesting that low-income students may exhibit lower academic engagement and more negative competitive attitudes. However, the current study found no significant difference in benign competitive attitude between low-income and non-low-income students. By contrast, low-income students scored significantly lower in malignant competitive attitude (*p* < 0.01). This discrepancy may be due to the higher professional accomplishments of non-low-income students’ parents, who often set elevated expectations and impose stricter academic demands on their children. Such parental pressure for success may drive non-low-income students to adopt extreme behavioral strategies in competitive contexts, thereby fostering malignant competitive attitudes.

### 4.3 Impact of single-parent status on parental rearing style and competitive attitude among low-income students

Existing studies indicate that single-parent families are disproportionately represented among low-income students, with their prevalence significantly higher than among non-low-income students (Toda et al., 2008). This study demonstrates that single-parent low-income students scored significantly higher in paternal emotional warmth and understanding (*p* < 0.05) but lower in maternal over-interference and overprotection (*p* < 0.05), maternal rejection and denial (*p* < 0.05), and maternal favoritism (*p* < 0.05) compared to non-single-parent low-income students (Hiko et al., 2023). Single-parent low-income students also exhibited lower benign competitive attitude scores (*p* < 0.05) (Ma et al., 2015). Potential explanations for these findings include: (1) Paternal Compensatory Behavior: Fathers in single-parent families may compensate for the absence of maternal care by providing heightened emotional warmth and understanding. (2) Maternal Stress and Parenting Patterns: Single mothers, burdened by socioeconomic and psychological pressures, may adopt restrictive, rejecting, or indulgent parenting behaviors. (3) Psychological Vulnerabilities: Children from single-parent families often experience low self-esteem, sensitivity, and depressive tendencies, which hinder cooperative social interactions and healthy competitive attitudes.

### 4.4 Correlation analysis between parental rearing style and competitive attitude among low-income students

This study reveals that parental emotional warmth and understanding significantly positively correlate with benign competitive attitude (*p* < 0.01) (Liu et al., 2020). Conversely, parental rejection and denial, favoritism, and overprotection show significant positive correlations with malignant competitive attitude (*p* < 0.05) (Asselmann et al., 2015). Notably, paternal punishment and strictness are also positively associated with malignant competitive attitude (*p* < 0.01) (Zhang et al., 2023). These findings suggest that parental emotional warmth fosters optimistic traits and adaptive communication skills, enabling students to engage constructively in competition. By contrast, critical, punitive, or dismissive parenting erodes self-esteem, leading to either competitive avoidance or excessive competitiveness through maladaptive strategies (Festen et al., 2013; Visser et al., 2013; Liber et al., 2008).

## 5 Conclusion

This study uncovers significant differences in parental rearing styles and competitive attitudes between low-income and non-low-income students. Family income indirectly shapes the competitive attitudes of students by influencing parental rearing practices. Consequently, universities should integrate ideological and political education with family education support for low-income students. For economically disadvantaged two-parent families, psychological counseling should be implemented to optimize parenting behaviors, foster harmonious parent–child relationships, and enhance family functioning. Concurrently, schools should provide necessary resources and support to assist parents in better understanding and addressing their children’s developmental needs. Special attention is required for single-parent households experiencing financial hardship. Parenting workshops should help single parents adopt more constructive disciplinary approaches while reducing excessive intervention in their children’s lives. Simultaneously, student mutual support groups should be established to facilitate emotional companionship among peers. Furthermore, gradual and structured competitive activities should be designed to help children develop positive mindsets toward competition within psychologically secure environments. Collectively, these measures support children from single-parent families in cultivating healthy perspectives on competition and building self-confidence within resource-constrained ecological conditions.

This study acknowledges certain limitations, notably that parental education level was not incorporated as a study variable. Future research should integrate parental educational background into analytical frameworks to comprehensively examine its interactive effects with family socioeconomic status on student behaviors. Such multidimensional analysis will advance a more holistic understanding of how familial environments shape psychological characteristics, thereby generating evidence-based guidance for family education practices.

## Data Availability

The original contributions presented in the study are included in the article/supplementary material, further inquiries can be directed to the corresponding author.

## References

[ref1] Afif R. (2019). Socioeconomic impacts: exploring relationships between parenting styles and emotional intelligence in ODD (Publication No. 6960). [Doctoral dissertation, Walden University]. ScholarWorks. Available online at: https://scholarworks.waldenu.edu/dissertations/6960

[ref2] Arnett J. J. (2000). Emerging adulthood: a theory of development from the late teens through the twenties. Am. Psychol. 55, 469–480. doi: 10.1037/0003-066X.55.5.469, PMID: 10842426

[ref3] Arnett J. J. (2014). Emerging adulthood: the winding road from the late teens through the twenties. 2nd Edn. Oxford: Oxford University Press.

[ref4] Asselmann E. Wittchen H. U. Lieb R. Höfler M. Beesdo-Baum K. (2015). The role of behavioral inhibition and parenting for an unfavorable emotional trauma response and PTSD. Acta Psychiatr. Scand. 131, 279–289. doi: 10.1111/acps.12316 25039395

[ref5] Boonk L. Gijselaers H. J. M. Ritzen H. Brand-Gruwel S. (2018). A review of the relationship between parental involvement indicators and academic achievement. Educ. Res. Rev. 24, 10–30. doi: 10.1016/j.edurev.2018.02.001

[ref6] Chen G. Li J. Lu F. (2003). Revision of the Chinese version of the competitive attitude scale. Psychol. Sci. 26, 332–333. doi: 10.3969/j.issn.1671-6981.2003.02.036

[ref7] Davis-Kean P. E. (2005). The influence of parent education and family income on child achievement: the indirect role of parental expectations and the home environment. J. Fam. Psychol. 19, 294–304. doi: 10.1037/0893-3200.19.2.294, PMID: 15982107

[ref8] Ding Y. Sun C. Dong B. (2024). Effect of parental rearing styles on adolescent ego identity: the mediating role of involutionary attitudes. Front. Psychol. 14:1292718. doi: 10.3389/fpsyg.2023.1292718, PMID: 38356993 PMC10866008

[ref9] Ding G. Xu L. Sun L. (2022). Association between parental parenting style disparities and mental health: an evidence from Chinese medical college students. Front. Public Health 10:841140. doi: 10.3389/fpubh.2022.841140, PMID: 35296043 PMC8918520

[ref10] Festen H. Hartman C. A. Hogendoorn S. de Haan E. Prins P. J. Reichart C. G.. (2013). Temperament and parenting predicting anxiety change in cognitive behavioral therapy: the role of mothers, fathers, and children. J. Anxiety Disord. 27, 289–297. doi: 10.1016/j.janxdis.2013.03.001 23602942

[ref11] Fontenot B. Uwayo M. Avendano S. M. Ross D. (2019). A descriptive analysis of applied behavior analysis research with economically disadvantaged children. Behav. Anal. Pract. 12, 782–794. doi: 10.1007/s40617-019-00389-8, PMID: 31976290 PMC6834796

[ref12] Ge M. Sun X. Huang Z. (2022). Correlation between parenting style by personality traits and mental health of college students. Occup. Ther. Int. 2022:6990151. doi: 10.1155/2022/6990151 (Retracted in Occupational Therapy International, 2024, 9872506, https://doi.org/10.1155/2024/9872506) 35936833 PMC9314183

[ref13] Hiko M. A. A. Es A. C. Baysen E. (2023). Single parenting and students’ academic performance. S. Afr. J. Educ. 43:2200. doi: 10.15700/saje.v43n4a2297

[ref14] Jeon S. Neppl T. K. (2016). Intergenerational continuity in economic hardship, parental positivity, and positive parenting: the association with child behavior. J. Fam. Psychol. 30, 22–32. doi: 10.1037/fam0000151, PMID: 26371448 PMC4742406

[ref15] Ju C. Wu R. Zhang B. You X. Luo Y. (2020). Parenting style, coping efficacy, and risk-taking behavior in Chinese young adults. J. Pac. Rim Psychol. 14:e24. doi: 10.1017/prp.2019.24

[ref16] Lee H. (2022). Family economic hardship and children’s behavioral and socio-emotional outcomes in middle childhood: direct and indirect pathways. Child Youth Serv. Rev. 113:105017. doi: 10.1016/j.childyouth.2022.106527

[ref17] Liber J. M. van Widenfelt B. M. Goedhart A. W. Utens E. M. van der Leeden A. J. Markus M. T.. (2008). Parenting and parental anxiety and depression as predictors of treatment outcome for childhood anxiety disorders: has the role of fathers been underestimated? J. Clin. Child Adolesc. Psychol. 37, 747–758. doi: 10.1080/15374410802359692 18991126

[ref18] Liu J. Peng P. Luo L. (2020). The relation between family socioeconomic status and academic achievement in China: a meta-analysis. Educ. Psychol. Rev. 32, 49–76. doi: 10.1007/s10648-019-09494-0

[ref19] Lundborg P. Nilsson A. Rooth D.-O. (2022). Parental education and offspring outcomes: evidence from the Swedish compulsory school reform. Am. Econ. Rev. 6, 253–278. Availabe at: http://www.jstor.org/stable/43189472

[ref20] Luo L. (2022). The practice of psychological well-being education model for poor university students from the perspective of positive psychology. Front. Psychol. 13:951668. doi: 10.3389/fpsyg.2022.951668, PMID: 35978785 PMC9376323

[ref21] Ma F. Evans A. D. Liu Y. Luo X. Xu F. Lee K. (2015). To lie or not to lie? The influence of parenting and theory-of-mind understanding on three-year-old children’s honesty. J. Moral Educ. 44, 198–212. doi: 10.1080/03057240.2015.1023182

[ref22] Masud H. Ahmad M. S. Cho K. W. Fakhr Z. (2019). Parenting styles and aggression among young adolescents: a systematic review of literature. Community Ment. Health J. 55, 1015–1030. doi: 10.1007/s10597-019-00400-0, PMID: 31102163

[ref23] Perris C. Jacobsson L. Linndström H. von Knorring L. Perris H. (1980). Development of a new inventory for assessing memories of parental rearing behaviour. Acta Psychiatr. Scand. 61, 265–274. doi: 10.1111/j.1600-0447.1980.tb00581.x, PMID: 7446184

[ref24] Rademacher A. Zumbach J. Koglin U. (2023). Parenting style and child aggressive behavior from preschool to elementary school: the mediating effect of emotion dysregulation. Early Child. Educ. J. 53, 63–72. doi: 10.1007/s10643-023-01560-1

[ref25] Toda M. Ezoe S. Nishi A. Mukai T. Goto M. Morimoto K. (2008). Mobile phone dependence of female students and perceived parental rearing attitudes. Soc. Behav. Personal. 36, 765–770. doi: 10.2224/sbp.2008.36.6.765

[ref26] Visser L. de Winter A. F. Vollebergh W. A. M. Verhulst F. C. Reijneveld S. A. (2013). The impact of parenting styles on adolescent alcohol use: the TRAILS study. Eur. Addict. Res. 19, 165–172. doi: 10.1159/000342558 23207481

[ref27] Yang J. Zhao X. (2020). Parenting styles and children’s academic performance: evidence from middle schools in China. Child Youth Serv. Rev. 113:105017. doi: 10.1016/j.childyouth.2020.105017

[ref28] Yue D. Li M. Jin K. Ding B. (1993). Preliminary revision of EMBU and its application in neurotic patients. Chin. Ment. Health J. 7, 97–101.

[ref29] Žerak U. Juriševič M. Pečjak S. (2024). Parenting and teaching styles in relation to student characteristics and self-regulated learning. Eur. J. Psychol. Educ. 39, 1327–1351. doi: 10.1007/s10212-023-00742-0

[ref30] Zhang H. Li S. Wang R. Hu Q. (2023). Parental burnout and adolescents’ academic burnout: roles of parental harsh discipline, psychological distress, and gender. Front. Psychol. 14:1122986. doi: 10.3389/fpsyg.2023.1122986, PMID: 36910745 PMC9995997

[ref31] Zhang W. Wei X. Ji L. Chen L. Deater-Deckard K. (2017). Reconsidering parenting in Chinese culture: subtypes, stability, and change of maternal parenting style during early adolescence. J. Youth Adolesc. 46, 1117–1136. doi: 10.1007/s10964-017-0664-x, PMID: 28357678

